# Remarks on Croup

**Published:** 1838-12-19

**Authors:** C. D. Meigs


### REMARKS ON CROUP. By C. D. Meigs, M. D.

(Continued from page 398.)

I think that the case of inflammatory croup, with tendency to plastic effusion, is one demanding the most prompt and resolute as well as the most judicious treatment. Considering the size of the tube of the larynx, which is barely sufficient for the admission of the quantity of air requisite for ordinary respiration, we are bound, in treating its inflammations, to take such measures as may, if possible, obviate any tendency to encroachment on its calibre. A very thin coating of lymph which obstinately adheres to it, serves to produce an evident difficulty of breathing—evident not only by the wheezing sound that accompanies it, but still more so by the length of the inspiration, which becomes protracted by the aid of the accessory muscles of respiration. How much more is the difficulty enhanced by a thick coating of lymph! The fatigue occasioned by the efforts to breathe with the aid of these muscles, soon expends the energies of the constitution; and the expression of the patient's countenance, which becomes pale, with eyes very open and staring, but somewhat sunken in the orbits, and a certain air of distress about the mouth and alæ of the nostrils, indicate the most imminent peril. The inflammation, which, in this case, may not occupy more than a couple of superficial inches, and which, were it seated upon the fauces, or cheeks, or stomach, would be of very little consequence, is here so fraught with danger and death, that it *must* be cured at once, if it can be.

What is the most powerful remedy that we can apply for the cure of the inflammation? The use of the lancet certainly offers us the most safe, sure, and easy remedy; but it is not merely to bleed the patient, that we ought to employ it—it is to be employed for the purpose of so far reducing the momentum of the blood in circulation, as to enable the inflamed vessels of the larynx to disgorge the blood with which they are injected into their collateral vessels, and which by injection tends to the production of a plastic effusion. I should never feel satisfied merely to reduce the momentum of the circulation sufficiently to bring it within moderate limits. I should always carry the bleeding so far as to make me feel assured, not only that the impetus would not again become great enough to maintain the high degree of inflammatory injection already existing, but that the whole of the secretions should be modified by the new rate of the circulation to be superinduced by the venesection. I should desire that, from the moment of the operation, the secretions of the larynx should begin to be thin and mucous, and to lose at once their plastic and albuminous, or fibrinous character, which is the cause of their cohesion to the mucous surface.

Hence the rule should be, where venesection is resorted to in croup, to carry it *usque ad deliquium.* If this is done, and well done, a very sensible relief is immediately obtained by the removal of all the spasm of the larynx which accompanies the inflammatory or plastic croup; and although the alleviation is not so striking as where a proper remedy is used in the merely spasmodic case of the malady, yet it is sufficiently so to afford encouragement and hope for the further application of remedies.

I am not a little fearful of the effects of tartar emetic, given immediately after an efficient venesection, in a young patient. Its relaxing power in some constitutions goes so far as to induce a state of real collapse, which is full of danger for the patient, and which, I doubt not, does sometimes prove fatal, in instances which might have a different issue upon the use of some less powerful medicine. The relaxation occasioned by a deliquium followed by the collapse resulting from the use of the antimonial emetic, is more than a very young constitution, not possessed of the greatest stamina, should be exposed to. On these accounts, I prefer the ipecacuanha. In a child six or eight years old, I should order ten grains of ipecacuanha, mixed with a teaspoonful of syrup of squills, and a tablespoonful of water. The dose is to be repeated in every quarter of an hour, until a full vomiting takes place.

The combination of squill and seneka, with tartar emetic, in the preparation so well known as Dr. Coxe's hive syrup, is certainly a very good one, and there is less danger of inducing the antimonial collapse by its use, than by that of the antimony alone. Nevertheless, as the ipecacuanha is capable of producing very full vomiting, with great nausea and most abundant discharges of fauceal mucus, I am inclined to look upon it as one of the drugs whose use is most clearly indicated for the occasion under review. I may add, that I think I have never seen a person injured or made ill by the use of ipecacuanha, whereas the employment of the antimonial is not unfrequently followed by the most alarming illness.

It is generally understood that the sympathy of the stomach with the fauces and throat is very active and sensible; and that upon account of this sympathetic relation, some patients in croup are with difficulty made to vomit, very large doses of tartar emetic, of antimonial wine, &c., being occasionally administered, only with the effect of producing a dangerous colliquation of the bowels, which reduces the strength with the rapidity occasioned by hæmorrhage itself. I have witnessed this effect of very large doses of antimonials. They are effects very much to be deprecated; and I have been accustomed, on this account, to make use of an emetic, which, so far as I can learn, is very little employed, but which, from its certainty and the speediness of its operation, ought to be more generally admitted to the list of available medicines for this particular case at least. I have been familiar with its effects for more than twenty years, and my confidence in them increases rather than diminishes by time.

In a child of two years, a teaspoonful of alum, either reduced to powder in a mortar, or scraped from a lump with a penknife, may be mixed with a teaspoonful of honey, and given as one dose, to be repeated in ten or fifteen minutes if vomiting does not take place. I think that I have never given more than two doses without causing very full vomiting; but I have often given large quantities of antimony, of antimonial wine, and ipecacuanha, without succeeding in exciting the efforts of the stomach. What further good effect this emetic may be capable of producing by its astringency, over and above its emetic power, I leave others to decide. I shall make no further remarks upon it, than that I should be well pleased if some of your readers would make trial of it upon proper occasion.

The warm bath, if the child be covered with water to the throat, is a remedy of great efficacy in the management of croup. It should be among the first things ordered, the more especially as it may be used at the same time with the others above mentioned; both the bleeding and the emetic may be administered while the patient is in the bath. If the water is of the temperature of from 94° to 96°, it will produce a redness of the surface, by inviting to the superficial vessels abundant supplies of blood, which have a derivative operation or influence upon the disorders of the throat.

The warm bath should be continued long enough to give a very decided determination of the circulation into the vessels of the skin, and it can be repeated several times, if the urgency of the symptoms point out the necessity for it.

(To be continued.)

